# Comparative analysis of environmental persistence of SARS-CoV-2 variants and seasonal coronaviruses

**DOI:** 10.1128/aem.01688-24

**Published:** 2025-03-06

**Authors:** Geun Woo Park, Boris Reija, Azaibi Tamin, Heather Hicks, Matthew Hayden Flanders, John M. Metz, Shufang Fan, Jennifer L. Harcourt, Jennifer M. Folster, Natalie Thornburg, Jan Vinjé

**Affiliations:** 1 Division of Viral Diseases, Centers for Disease Control and Prevention 1242 , Atlanta, Georgia, USA; 2 Cherokee Nation Assurance, Arlington, Virginia, USA; 3 Coronavirus and Other Respiratory Viruses Division (CORVD), Centers for Disease Control and Prevention 1242 , Atlanta, Georgia, USA; 4 ASRT, Inc, Atlanta, Georgia, USA; 5 Division of Core Laboratory Services and Response (DCLSR), Centers for Disease Control and Prevention 1242 , Atlanta, Georgia, USA; University of Nebraska-Lincoln, Lincoln, Nebraska, USA

**Keywords:** SARS-CoV-2, SARS-CoV-2 surrogate, OC43, bovine coronavirus, seasonal coronaviruses, environmental persistence

## Abstract

Conducting persistence studies of infectious severe acute respiratory syndrome coronavirus 2 (SARS-CoV-2) on environmental surfaces may require a biosafety level 3 (BSL-3) laboratory. We aimed to compare the environmental persistence of BSL-2 level human coronaviruses (229E, NL63, and OC43) and bovine coronavirus (BoCoV) with three SARS-CoV-2 variants (WA-1, Delta, and Omicron). OC43 (1.8 TCID_50_/mL) and BoCoV (1.0 TCID_50_/mL) had lower detection thresholds in cell culture assays compared to 229E (150 TCID_50_/mL) and NL63 (2,670 TCID_50_/mL) and were used for persistence tests at room temperature. Viable OC43 became undetectable (>5.2log_10_) after 48 hours on stainless steel and plastic coupons but exhibited extended persistence up to 72 hours on touchscreen glass coupons. In contrast, BoCoV remained viable for up to 120 hours with <1.8 log_10_ infectivity loss. Both OC43 and BoCoV showed a reduction of >5 log_10_ on vinyl coupons after 48 hours. On stainless steel coupons, the viability of all three SARS-CoV-2 variants became undetectable (>2.3 log_10_ reduction) after 48 hours, with minor differences in reduction levels at 24 hours, whereas on touchscreen glass coupons, the viable virus could be detected for up to 48 hours for WA-1 and Omicron and 72 hours for the Delta variant. Regardless of coupon or virus type, viral RNA titers increased <4.5 Ct values after 120 hours. Our data demonstrate distinct persistence characteristics between BoCoV and OC43, with neither fully mimicking SARS-CoV-2 variants. This variability along with the impact of surface types on viral persistence underscores the need for caution when using these viruses as surrogates for SARS-CoV-2.

**IMPORTANCE:**

In this study, we evaluated three human seasonal coronaviruses (OC43, NL63, and 229E) and one bovine coronavirus (BoCoV) as potential surrogate viruses for SARS-CoV-2. Our data suggest that among the four surrogate viruses tested, OC43 and BoCoV were the most promising candidates due to their assay sensitivity, ease of handling, and high genetic similarity to SARS-CoV-2. However, neither BoCoV nor OC43 fully mimicked the environmental persistence characteristics of SARS-CoV-2 variants highlighting the potential limitations of using surrogate viruses.

## INTRODUCTION

SARS-CoV-2, the virus causing COVID-19, is a novel positive-sense single-stranded RNA virus that belongs to the genus *Betacoronavirus* of the *Coronaviridae* family (1, 2). This genus also encompasses other highly pathogenic coronaviruses (CoVs) including SARS-CoV, responsible for the 2002–2003 SARS epidemic, and the virus responsible for the Middle East respiratory syndrome CoV (1, 2). The principal transmission modes for SARS-CoV-2 are direct contact, droplet, and airborne (3). Although not a primarily transmission route, surfaces can become contaminated with the virus through contact with respiratory droplets or contaminated hands from infected individuals, and therefore, surfaces are considered a potential reservoir for the spread of SARS-CoV-2 in semi-closed settings such as households, cruise ships, and hospitals (4–7).

To develop effective public health prevention strategies against SARS-CoV-2 transmission, such as effective cleaning protocols, understanding virus survival characteristics on environmental surfaces is crucial. While the World Health Organization (WHO) has identified SARS-CoV-2 as a Risk Group (RG) 2 pathogen suitable for handling in a BSL-2 laboratory (8), the US continues to treat it as an RG3. This requires BSL-3 containment for all laboratory work, especially when working with high-titer viral isolates, which presents significant technical and financial challenges (9). Hence, the use of surrogate viruses with similar physio-chemical properties as SARS-CoV-2 that can be studied in BSL-2 laboratories has been considered (9). Because of their genetic similarity and transmission modes akin SARS-CoV-2, several members of the *Coronaviridae* family, such as 229E, NL63, and OC43, have been considered as suitable surrogates (1–3). Data derived from these viruses have been used to understand physicochemical properties and transmission modes of SARS-CoV-2 (3, 10). Additionally, coronaviruses of animal origin such as bovine coronavirus (BoCoV), porcine epidemic diarrhea virus, phi6 bacteriophage, and murine hepatitis virus (MHV), which pose less exposure risk to researchers, have also been evaluated as potential surrogate viruses for SARS-CoV-2 (11–17).

However, comparing data from different studies is challenging, primarily because varying test conditions, such as temperature, humidity, and surface type, have been used (11, 12, 15, 18–21). Moreover, cell culture assays, which are pivotal for measuring viable virus, require cell lines that produce virus-induced cytopathic effect (CPE) that can be easily measured by 50% tissue culture infective dose (TCID_50_) or plaque assays (1, 22–24). In addition to the use of different cell lines, the difference in time (ranging from 4 to 7 days) to confirm complete CPE (18, 21, 22, 24, 25) makes it challenging to compare data from different studies. In the current study, we compared the environmental persistence of an endemic human coronavirus (OC43) and BoCoV with three major SARS-CoV-2 variants (WA-1, Delta, and Omicron) on different hard surfaces using cell culture infectivity and viral RNA persistence as read-out.

## MATERIALS AND METHODS

### Viruses and cell lines

Human coronavirus 229E (ATCC VRL-740), NL63 (ATCC NR-470), and OC43 (VR-1558) were purchased from ATCC (Manassas, VA), and BoCoV (NR- 445) was obtained from BEI resources (Manassas, VA). SARS-CoV-2 variants used in this study included the Wuhan strain, the original strain of SARS-CoV-2, and two other global variants (Delta and Omicron) (26–28). Three SARS-CoV-2 strains (WA-1, Delta [B.1.617.2], and Omicron [BA.5.2.1]) were used in this study. WA-1 is the US ancestral SARS-CoV-2 variant that was reported previously (26), and both the Delta (B.1.617.2; GenBank accession #: MZ082533) and Omicron (BA.5.2.1; GenBank accession #: OP292338) variants were isolated from clinical specimens collected during SARS-CoV-2 outbreaks in the US in 2022. The strains were exempt from IRB review (CDC-NCIRD-VGB-6/17/24-c7571) as the research involved de-identified, unlinkable biospecimens and did not include Food and Drug Administration (FDA) investigational products. The following cell lines were obtained from ATCC and BEI resources (Manassas, VA) and used to propagate/assay the respective viruses: MRC-5 (CCL-171, ATCC), LLC MK2 (CCL-7, ATCC), HCT-8 (CCL-244, ATCC), HRT-18G, a derivative cell from HCT-8 (CRL-11663, ATCC) and Vero E6/TMPRSS2-T2A-ACE2 (NR-54970, BEI resources). All cell lines were cultured at 37°C and 5% CO_2_ using their specific individual cell culture medium (Table 1).

**TABLE 1:** Overview of cell lines and culture media used in this study

Cell line^*a*^	Source	Medium composition^*b*^
MRC-5	Human lung epithelial cell	DMEM + 10% FBS, 1% pen/strep,1% L-glutamine, 1% HEPES, and 1% sodium pyruvate
LLC-MK2	Rhesus monkey kidney	Opti-MEM + 2% FBS, and 1% pen/strep
HCT-8	Human ileocecal adenocarcinoma cell	DMEM + 3% FBS, 1% pen/strep, and 1% sodium pyruvate
HRT-18G	Subclone of HRT-18	DMEM + 3% FBS, 1% pen/strep, and 1% sodium pyruvate
Vero E6/TMPRSS2-T2A-ACE2	Vero E6 recombinant cell line	DMEM + 10% FBS, 2% pen/strep, puromycin, and amphotericin B

^*a*^ Purchased from ATCC (Manassas, VA, USA).

^*b*^ Dulbecco’s modified Eagle medium (DMEM), fetal bovine serum (FBS), sodium pyruvate (100 mM), L-glutamine (200 mM), HEPES (1 M), penicillin-streptomycin (pen/strep, 10,000 U/mL), and puromycin (10 mg/mL). All products were purchased from Thermo Fisher Scientific (Waltham, MA, USA).

### Coupons of different environmental surfaces

Vinyl fabric (Mayer-Paetz Fabrics [Indianapolis, IN]) and plastic sheets (ePlastics [San Diego, CA]) were manually cut into 1 × 1 cm^2^ coupons. Stainless steel (1 × 1 cm^2^) and touch screen glass coupons (1 × 1 cm^2^) were purchased from Muzeen & Blythe Ltd (Winnipeg, Canada) and Valley design Corp (Shirley, MA). Prior to use, all coupons were cleaned with soap, rinsed in 70% ethanol, and air dried. Stainless and vinyl fabric coupons were wrapped in aluminum foil and autoclaved for 15 minutes at 121°C, while plastic and touchscreen glass coupons were disinfected by cleaning with 70% ethanol and then exposed to UV irradiation in a biosafety cabinet for 10 minutes. All sterilized coupons were stored in sterile 50 mL Falcon tubes until use.

### Virus propagation

All viruses were propagated as previously described (18, 26, 29). Specifically, a T-75 flask containing confluent cell monolayers was inoculated with each virus at a multiplicity of infection of 0.01–0.001 in 5 mL cell culture medium and incubated for 1.5 hours at 33°C and 5% CO_2_ (Table 2) with the flask being manually shaken every 30 minutes. The virus inoculum was discarded and replaced with 25 mL of infection medium (Table 2), and the cell monolayers were incubated at 33°C with 5% CO_2_ for up to 9 days for OC43 and BoCoV or up to 14 days for NL63 and 229E. Cell monolayers were monitored daily until over 50% of cells showed a cytopathic effect. After subjecting the flasks to three times freeze/thaw cycles, cell debris was removed by centrifugation at 800 × *g* for 20 minutes. The clarified supernatant was aliquoted and stored at −80°C until use.

**TABLE 2:** Overview of virus culture media used in this study

Strain^*a*^	Propagated in	Medium composition^*b*^
229	MRC-5, 33°C	DMEM + 5% FBS, 1% pen/strep, 1% L-glutamine, 1% HEPES, and 1% sodium pyruvate
NL63	LLC-MK2, 33°C	Opti-MEM and 1% pen/strep
OC43	HCT-8 and HRT-18G, 33°C	DMEM + 2% FBS, 1% pen/strep, and 1% sodium pyruvate
BoCoV	HRT-18G, 33°C	DMEM + 2% FBS, 1% pen/strep, and 1% sodium pyruvate
SARS-CoV-2 variants^*c*^	Vero E6/TMPRSS2-T2A-ACE2, 37°C	DMEM + 10% FBS, 2% pen/strep, puromycin, and amphotericin B

^*a*^ Purchased from ATCC (Manassas, VA, USA).

^*b*^ Dulbecco’s modified Eagle medium (DMEM), penicillin-streptomycin (pen/strep, 10,000 U/mL), fetal bovine serum (FBS), sodium pyruvate (100 mM), L-glutamine (200 mM), HEPES (1 M), amphotericin B (250 µg of amphotericin B and 205 µg of sodium deoxycholate/mL) and puromycin (10 mg/mL). All products were purchased from Thermo Fisher Scientific (Waltham, MA, USA).

^*c*^ SARS-CoV-2 WA-1, Delta, and Omicron.

### Virus titration

Each virus stock was titrated using 10-fold serial dilutions to determine the TCID_50_ on 96-well culture plates with slight modifications from the original protocols (22–24). The 96-well plates were incubated for up to 2 weeks at 33°C and 5% CO_2_, or until over 50% of cells showed a cytopathic effect. For SARS-CoV-2 variants, 96-well plates were incubated for up to 1 week at 37°C and 5% CO_2_. Subsequently, CPE in each well was scored as positive or negative, and TCID_50_ titers were calculated from four replicates (surrogate viruses) or ten replicates (SARS-CoV-2 variants) using the improved–Kärber Method (30). The limit of detection was 5.6 (2.1–15.0) TCID_50_/mL for the surrogate viruses and 125.9 (62.5–254) TCID_50_/mL for SARS-CoV-2 variants. Stock titers were 1.0 × 10^6^ TCID_50_/mL for 229E, 1.8 × 10^7^ TCID_50_/mL for NL63, 3.8 × 10^7^ TCID50/mL for OC43, 1.8 × 10^8^ TCID_50_/mL for BoCoV, 1.4 × 10^7^ TCID_50_/mL for WA-1, 2.8 × 10^7^ TCID_50_/mL for Delta (B.1.6.617.2), and 2.8 × 10^7^ TCID_50_/mL for Omicron (BA.5.2.1).

### Detection of coronaviruses by TaqMan real-time RT-PCR

Viral RNA was extracted from 50 µL of viral suspension using the MagMAX-96 Viral RNA Isolation Kit (Ambion, Austin, TX) on an automated KingFisher extractor according to the manufacturer’s instructions. SARS-CoV-2 variants that were eluted from coupons in a biosafety cabinet in a BSL-3 laboratory were inactivated by adding MagNA Pure 96 external lysis buffer (Roche Diagnostics, Indianapolis, IN) and subsequently transferred to a BSL-2 lab to complete viral RNA extraction. Extracted viral RNA samples were then tested by virus-specific real-time RT-PCR (RT-qPCR) assays using the AgPath-ID One-Step RT-PCR Kit (ThermoFisher Scientific) on an ABI 7500 platform. Oligonucleotide primer and probe information for each virus-specific RT-qPCR assay (229E, OC43, NL63, BoCoV, and SARS-CoV-2 variants) were obtained from previous studies (31–33) and is summarized in Table 3. For seasonal coronaviruses, the PCR reaction conditions included an initial reverse transcription step at 45°C for 10 minutes (one cycle), followed by 95°C for 10 minutes (one cycle), and then 40 cycles of 95°C for 15 seconds and 50°C for 1 minute for 229E, NL63, and OC43, or 55°C for 1 minute for BoCoV, with slight modifications from original studies (31, 32). The RT-qPCR assay to detect SARS-CoV-2 variants was a multiplex PCR assay targeting SARS-CoV-2 and influenza viruses A and B (33). Reaction conditions for the multiplex RT-qPCR assay included an annealing step at 25°C for 2 minutes, a reverse transcription step at 50°C for 15 minutes, Taq activation at 95°C for 2 minutes, followed by 45 cycles of 95°C for 15 seconds and 55°C for 30 seconds (33).

**TABLE 3:** Information on primers and probes used in this study

Strain	Primer/probe	Gene target	Sequence 5′−3′	Reference
229	N229E-1	Nucleoprotein	CAG TCA AAT GGG CTG ATG CA	31
	N229E-2		AAA GGG CTA TAA AGA GAA TAA GGT ATT CT	
	N229E-p^*a*^		CCC TGA CGA CCA CGT TGT GGT TCA	
NL63	HuNL63-1	Nucleoprotein	GAC CAA AGC ACT GAA TAA CAT TTT CC	31
	HuNL63-2		ACC TAA TAA GCC TCT TTC TCA ACC C	
	HuNL63-p^*a*^		AAC ACG CT"T"^*b*^ CCA ACG AGG TTT CTT CAA CTG AG	
OC43	NOC43-1	Nucleoprotein	CGA TGA GGC TAT TCC GAC TAG GT	31
	NOC43-2		CCT TCC TGA GCC TTC AAT ATA GTA ACC	
	NOC43-p^*a*^		TCC GCC TGG CAC GGT ACT CCC T	
BoCoV	BCoV-F	Transmembrane (M) gene	CTG GAA GTT GGT GGA GTT	32
	BCoV-R		ATT ATC GGC CTA ACA TAC ATC	
	BCoV-P^*a*^		CCT TCA TAT CTA TAC ACA TCA AGT TGTT	
SARS-CoV-2 variants^*c*^	Forward	Nucleocapsid protein	CTG CAG ATT TGG ATG ATT TCT CC	33
	Reverse		CCT TGT GTG GTC TGC ATG AGT TTA G	
	Probe^*a*^		ATT GCA ACA TAO ATC CAT GAG CAG TGC TGA CTC	

^*a*^ Probes are labeled at the 5′-end with the reporter molecule 6-carboxyfluorescein (FAM) and at the 3′-end with the quencher Black hole quencher-1.

^*b*^ Labeled at the 5′-end with FAM, internally quenched with Black hole quencher-1 (indicated by “T”), and labeled at the 3′-end with a phosphate.

^*c*^ Multiplex real-time reverse transcription PCR for influenza A virus, influenza B virus, and severe acute respiratory syndrome coronavirus.

### Sensitivity of cell culture assays

To assess the sensitivity of the cell culture assays of the surrogate viruses, we examined the relationship between viral replication in host cells and CPE manifestation, with a slight modification of the protocols used previously (34, 35). Specifically, monolayers of the different cell lines (Table 2) for 229E, NL63, OC43, and BoCoV were prepared in 96-well plates and incubated at 33°C and 5% CO_2_ for 1 week. Each virus stock was serially diluted 5- or 10-fold in infection media to obtain virus concentrations ranging from 10^0^ to 10^6^ TCID_50_/mL.

On day 0, cell monolayers with a confluence greater than 90% were infected with 100 µL of each virus dilution in four wells and incubated at 33°C and 5% CO_2_ for 7 days. One plate was randomly selected at days, 3, 4, 5, 6, and 7, and cells were monitored for CPE. At day 7, virus dilutions that exhibited both wells with and without CPE and up to two subsequent dilutions were extracted using a 96-well KingFisher instrument after 3× freeze thawing and tested by virus-specific RT-qPCR.

One plate was designated as a reference plate (T_ref_) to determine the basal RNA level of each virus at 0 hour. Cell lysates harvested immediately after virus inoculation served as the zero-time point sample. Comparatively, lysates harvested at specified time points acted as T time point samples. Viruses in cell culture lysates with a decrease of more than 3.3 Ct values between inoculation and the respective sampling time (Ct _*t* = *t* day_) (Ct_*t* = 0_ − Ct_*t* = *t* day_) indicated the presence of replicative viruses. We determined the assay sensitivity for each virus by comparing the earliest time points at which the presence of replicative viruses was indicated, and CPE was observed across different inoculum concentrations.

### Virus persistence on stainless steel, plastic, vinyl, and touchscreen glass coupons

Stainless steel and touchscreen glass (the primary surface material of mobile phone) were chosen for this testing as they are highly representative of frequently touched surfaces. Twenty microliters of OC43 (10^7^ TCID_50_/mL) or BoCoV (10^7^ TCID_50_/mL) in Dulbecco’s modified Eagle medium (DMEM) supplemented with 2% FBS was applied in triplicate onto coupons of stainless steel, plastic, vinyl, and touchscreen glass. The titer of each SARS-CoV-2 variant was diluted 10-fold to 10^6^ TCID_50_/mL using DMEM supplemented with 2% FBS and applied in triplicate to stainless steel and touchscreen glass coupons. Corresponding Ct values for virus inoculums of WA-1, Delta (B.1.617.2), and Omicron were 27.0, 26.4, and 28.0. All inoculated coupons were then left to dry in a biosafety cabinet at room temperature. We eluted the virus immediately after deposition and verified that initial viral titers remained consistent throughout the study, confirming that the elution procedure alone did not inactivate the virus. These data served as a baseline, and all viral reductions at different time points were calculated by subtracting the remaining infectious titers at each time point from those at time zero. At various post-inoculation (PI) times (6, 24, 48, 72, and 120 hours), the virus was eluted from each coupon in 1 mL of infection medium by pipetting up and down for 15 seconds and stored at −80°C until further analysis.

### Infectivity assay of eluted viruses from coupons

The viability of the eluted viruses was tested as follows. HRT-18 G cells (ATCC CRL-11663) for culturing OC43 and BoCoV or Vero E6/TMPRSS2-ACE2 for SARS-CoV-2 strains (WA-1, Delta [B.1.617.2] and Omicron [BA.5.2.1]) were seeded in 96-well plates and incubated at 37°C and 5% CO_2_ for 1 week when they formed monolayers. Coupon eluants were 10-fold serially diluted in DMEM media supplemented with 2% FBS and inoculated on four 96-wells with cell monolayers and incubated in a CO_2_ incubator at 33°C (OC43 and BoCoV) or 37°C (SARS-CoV-2 variants) for 7 days. Plates were microscopically examined for virus-induced CPE, and titers were calculated using improved–Kärber Method and reported as TCID_50_/mL (30). Additionally, viral RNA was extracted from all coupon eluates and assayed by RT-qPCR to assess the stability of viral RNA over time.

### Statistical analysis

The titer reduction level on the coupon post-inoculation was determined by calculating the log_10_ (Nt/N_0_), where N_0_ represents the viable virus count on coupons immediately post-inoculation, and Nt denotes the titer of viable viruses recovered from coupons T hours post-inoculation. Data were obtained from at least three samples, averaged, and subjected to statistical analysis. Group differences in log_10_ virus reduction, based on test variables (coupon or virus type), were assessed using either the Mann-Whitney *U*-test or the Kruskal-Wallis test, as appropriate. Analyses were conducted using PASW Statistics, version 21 (36). *P* values of < 0.05 were considered statistically significant.

## RESULTS

We tested multiple cell lines for culturing 229E, OC43, NL63, and BoCoV. Infectious titers (corresponding Ct value) ranged from 1.5 × 10^4^–1.5 TCID_50_/mL (23.8–33.9; 229E), 2.7 × 10^4^–2.7 TCID_50_/mL (25.9–34.7; NL63), 1.8 × 10^4^–1.8 TCID_50_/mL (21.8–36.5; BoCoV), and 1.0 × 10^4^–1.0 TCID_50_/mL (16.4–27.3; OC43). Viral replication was detected by RT-qPCR in MRC-5 cells infected with 229E at concentrations of 1.5 TCID_50_/mL at day 3 PI (Table 4). In contrast, incubation for 7 days was needed to confirm CPE in MRC-5 cells infected with 229E at 150 TCID_50_/mL. At day 3 post-inoculation, LLC-MK-2 cells infected with the minimum concentration of 2.7 TCID_50_/mL of NL63 showed replication, as detected by RT-qPCR. However, CPE was not observed in cells inoculated at concentrations of <2,670 TCID_50_/mL until day 7 PI. Overall, despite the presence of replication in MRC-5 and LLC-MK2 cells inoculated with 229E and NL-63 after a 3-day incubation period, 229E and NL63 at titers of <150 and 2,670 TCID_50_/mL did not exhibit CPEs within a 7-day incubation period. This limitation restricts the sensitivity of the cell culture assays in confirming the presence of lower-titer 229E and NL63, and therefore, stability on coupons was not further investigated in these viruses.

**TABLE 4:** Detection limit of cell culture assays for four coronaviruses

Strain	Cell line	Minimum viral concentration required to manifest viral infectivity			
		Infectivity marker	Viral concentration		Period of incubation^*c*^
			Infectivity (TCID_50_/mL)	Corresponding Ct value	
229E	MRC-5	Viral replication^*a*^	1.5	25.7 ± 0.3	≤3
		CPE^*b*^	150	21.2 ± 0.1	7
NL63	LLC-MK2	Viral replication	2.7	32.5 ± 0.6	≤3
		CPE	2,670	26.8 ± 0.1	7
OC43	HCT-8	Viral replication	1.0	27.4 ± 0.1	≤3
		CPE	10.0	25.6 ± 0.0	7
	HRT-18G	Viral replication	1.0	27.4 ± 0.1	≤3
		CPE	1.0	27.4 ± 0.1	6
BoCoV	HRT-18G	Viral replication	1.8	36.5 ± 0.0	≤3
		CPE	1.8	36.5 ± 0.0	5

^*a*^ RNA samples extracted from these cell lysate suspensions were subsequently tested by real-time RT-PCR assay targeting the respective viruses. For reference, cell lysate harvested immediately after virus inoculation served as the zero-time point sample. Viruses in cell culture lysates with a decrease of more than 3.3 Ct values between the zero-time point and the respective sampling time indicate the presence of replicative viruses.

^*b*^ Cytopathic effects (CPE).

^*c*^ Incubation time required for CPE in over 50% of infected cells.

**Fig 1:**
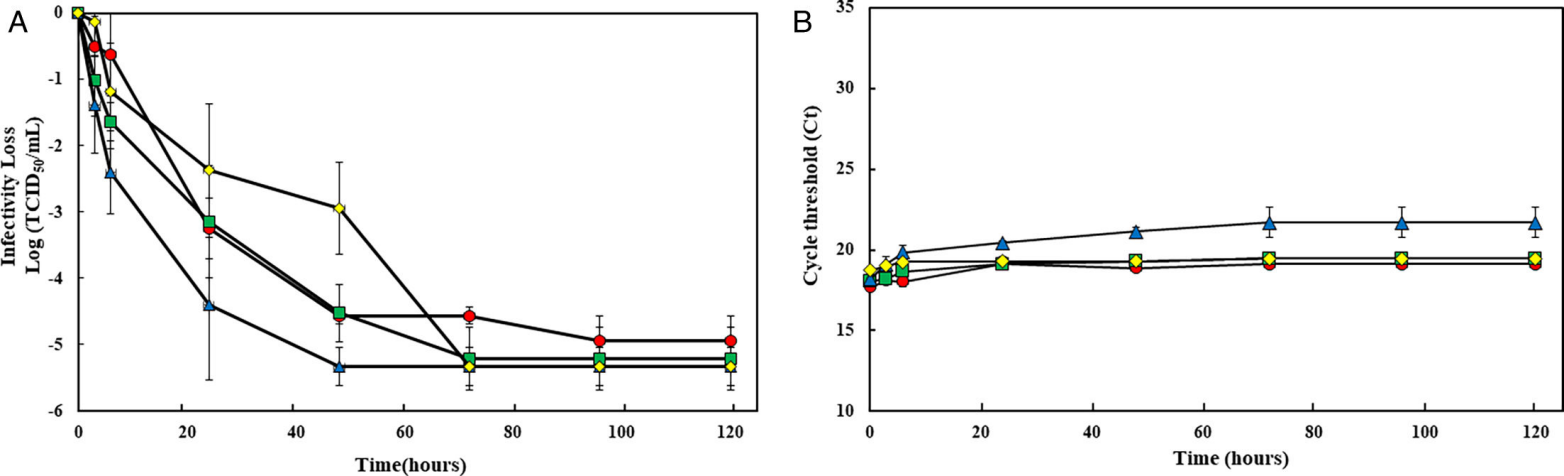
Survival of human coronavirus OC43 on four types of surface coupons. Twenty microliters of virus (OC43) with an infectious titer of approximately 10^7^ TCID_50_/mL were deposited on each coupon (stainless [○, filled in red], plastic [□, filled in green], vinyl [Δ, filled in yellow], and touchscreen glass [◊, filled in blue]) in quadruplicate and left to dry in a biosafety cabinet (BSC) at room temperature. At post -inoculation times of 0, 3, 6, 24, 48, 72, 96, and 120 hours, the virus was eluted from the coupon by pipetting with 1 mL of infection media (DMEM plus 2% FBS). Eluted samples were then tested by cell culture, followed by an RT-qPCR assay to confirm viral infectivity (A). Also, eluted samples were RNA extracted and assayed by RT-qPCR assay to measure residual RNA (B). Each data point is the mean of at least six replicates, with error bars showing the SD.

HCT-8 cells inoculated with 1.0 TCID_50_/mL OC43 showed viral replication by RT-qPCR at day 3 PI, whereas CPE began manifesting by day 7 PI at ≥10.0 TCID_50_/mL OC43. No CPE was evident in cells with <10.0 TCID_50_/mL even by day 7 PI. HRT-18G cells infected with 1.0 TCID_50_/mL OC43 exhibited viral replication by RT-qPCR at day 3 PI, while CPE was observed by day 6 PI. HRT-18G cells infected with ≥1.8 TCID_50_/mL BoCoV showed viral replication by RT-qPCR at day 3 PI and CPE by day 5 PI. Overall, HRT-18G cells exhibited CPE by days 6 and 5 post-infection at 1.0 and 1.8 TCID₅₀/mL for OC43 and BoCoV, respectively, indicating a more rapid manifestation of CPE in response to OC43 compared to HCT-8 cells. Using titration of eluted virus, we tested the environmental stability of OC43 on different surfaces. The titer of OC43 was reduced by more than 2.0 logs after 24 hours on all surfaces. After 48 hours, some differences were observed with an additional reduction of OC43 of 2.9 log reduction on glass, 4.5 log reduction on stainless steel and plastic coupons (*P* = 0.046), and >5.1 log on vinyl coupons. However, regardless of the coupon type, OC43 was inactivated to below the detection limit after 72 hours (Fig. 1A). In contrast, the viral RNA titers of OC43 on all four different coupons remained constant up to 120 hours PI (Fig. 1B).

**Fig 2:**
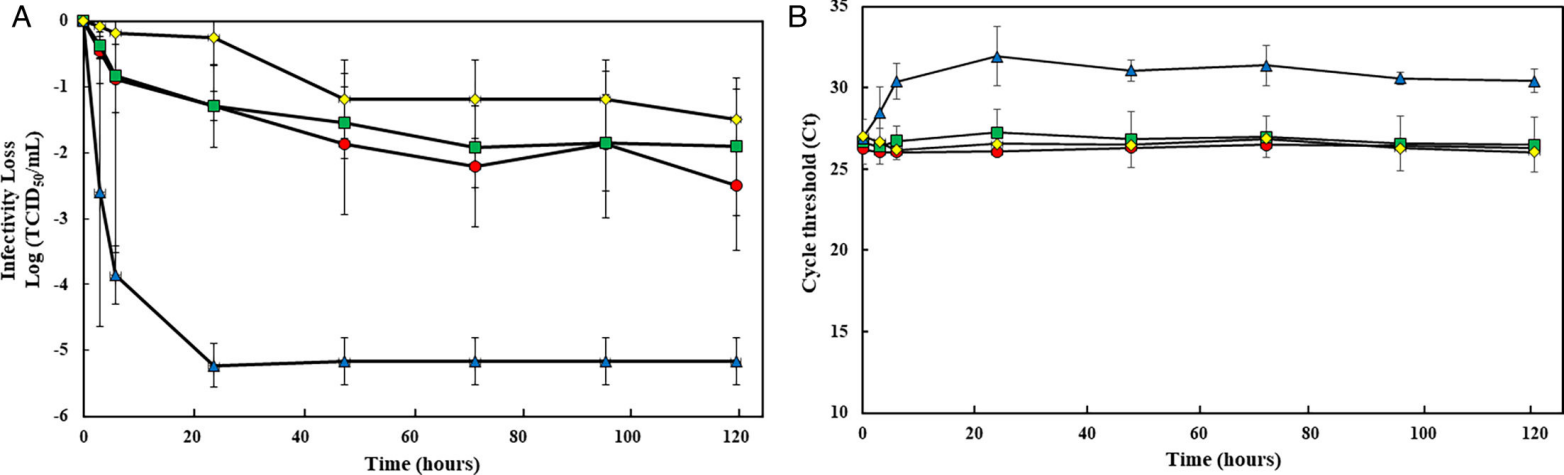
Survival of BoCoV on four types of surface coupons. Twenty microliters of BoCoV with an infectious titer of approximately 10^7^ TCID_50_/mL were deposited on each coupon (stainless [○, filled in red], plastic [□, filled in green], vinyl [Δ, filled in yellow], and touchscreen glass [◊, filled in blue]) in quadruplicate and left to dry in a BSC at room temperature. At post -inoculation times of 0, 3, 6, 24, 48, 72, 96, and 120 hours, the virus was eluted from the coupon by pipetting with 1 mL of infection media (DMEM plus 2% FBS). Eluted samples were then tested by cell culture, followed by an RT-qPCR assay to confirm viral infectivity (A). Also, eluted samples were RNA extracted and assayed by RT-qPCR assay to measure residual RNA (B). Each data point is the mean of at least six replicates, with error bars showing the SD.

Compared to OC43, BoCoV lost infectivity more slowly on steel, plastic, and glass coupons with a reduction of titers ranging from 1.2 to 1.8 log TCID50/mL after 120 hours. The levels of BoCoV reduction on glass coupons were consistently lower compared to steel and plastic coupons. In contrast, on vinyl coupons, the titer reduced by more than 2 log within 3 hours, and no infectious virus was detectable after 48 hours (Fig. 2A). The Ct values of BoCoV RNA on vinyl coupons increased by 5.0 after 120 hours while remaining the same for the other three coupon types (Fig. 2B).

When comparing the persistence of OC43 and BoCoV across different coupon types, both viruses showed distinct persistence patterns on stainless steel, plastic, and glass coupons with BoCoV demonstrating greater stability than OC43 (*P* < 0.01). However, on vinyl coupons, there was no significant difference between OC43 and BoCoV (*P* = 0.235; Fig. 1 and 2).

**Fig 3:**
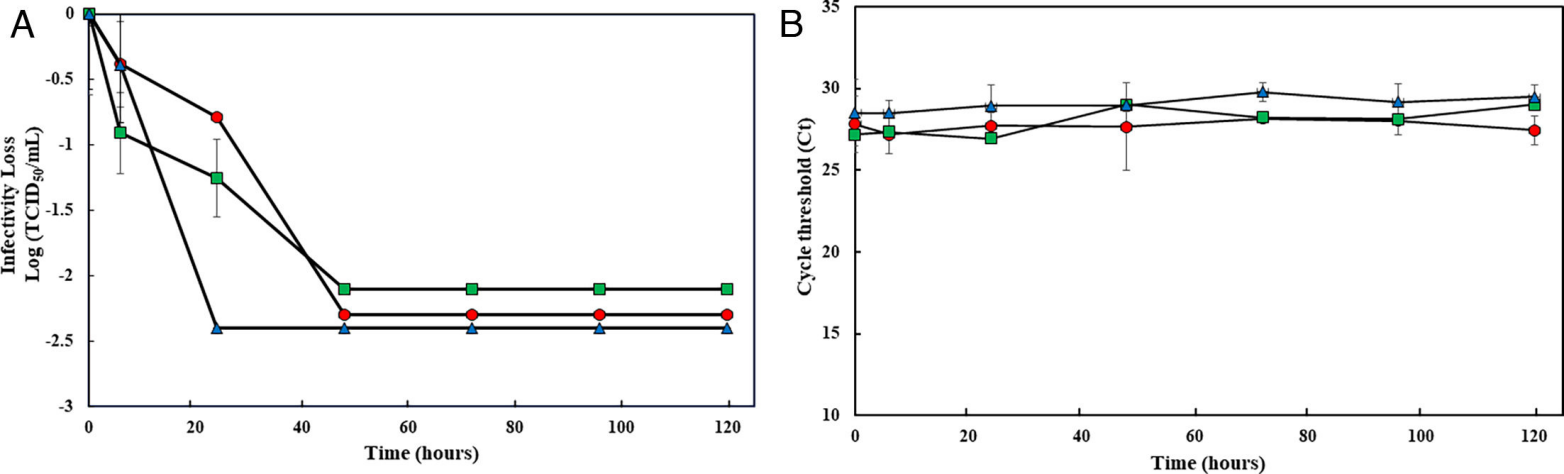
Survival of three SARS-CoV-2 variants (WA-1, Delta, and Omicron) on stainless steel. Twenty microliters of virus (SARS-CoV-2 WA-1 [○, filled in red], Delta [□, filled in green], and Omicron [Δ, filled in blue]), with an infectious titer of approximately 10^6^ TCID_50_/mL, were deposited on stainless steel in quadruplicate and left to dry in a BSC at room temperature. At post-inoculation times of 0, 6, 24, 48, 72, 96, and 120 hours, the virus was eluted from the coupon by pipetting with 1 mL of infection media (DMEM plus 2% FBS). Eluted samples were then tested by cell culture to determine infectious virus titers (A). Also, eluted samples were RNA extracted and assayed by RT-qPCR assay to measure residual RNA (B). Each data point is the mean of 3–4 replicates, with error bars showing the SD.

**Fig 4:**
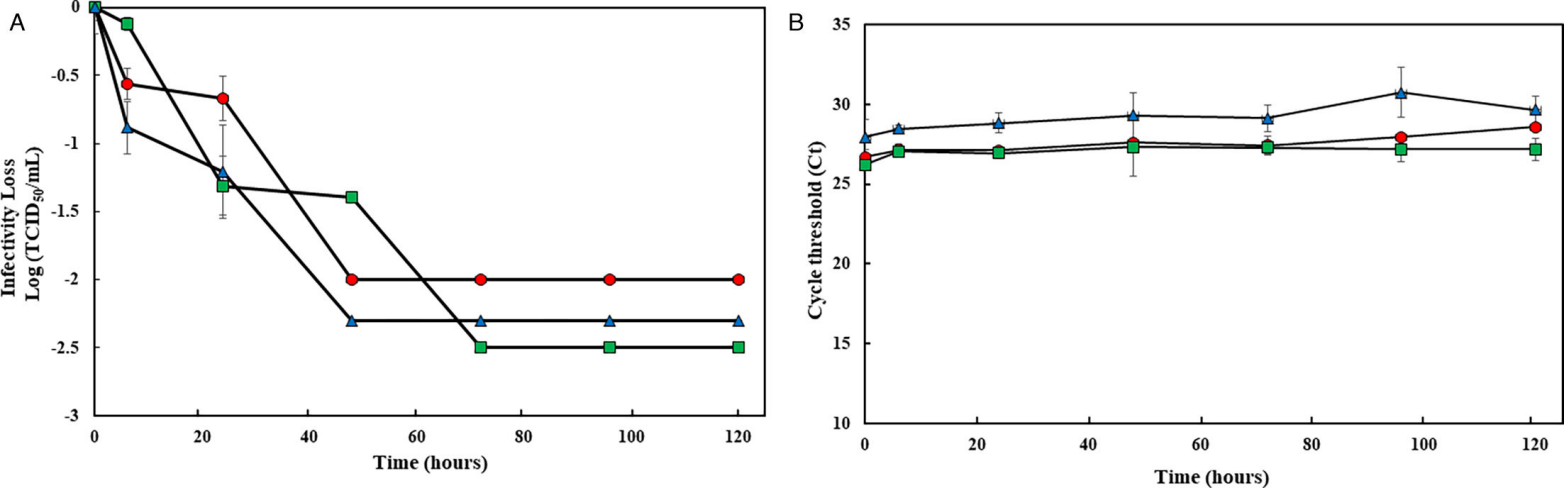
Survival of three SARS-CoV-2 variants (WA-1, Delta, and Omicron) on touchscreen glass coupons. Twenty microliters of virus (SARS-CoV-2 WA-1 [○, filled in red], Delta □, filled in green), and Omicron [Δ, filled in blue]), with an infectious titer of approximately 10^6^ TCID_50_/mL, were deposited on touchscreen glass coupons in quadruplicate and left to dry in a BSC at room temperature. At post-inoculation times of 0, 6, 24, 48, 72, 96, and 120 hours, the virus was eluted from the coupon by pipetting with 1 mL of infection media (DMEM plus 2% FBS). Eluted samples were then tested by cell culture to determine infectious virus titers (A). Also, eluted samples were RNA extracted and assayed by RT-qPCR assay to measure residual RNA (B). Each data point is the mean of 3–4 replicates, with error bars showing the SD. Each data point is the mean of 3–4 replicates, with error bars showing the SD.

The patterns of loss of viability of the three SARS-CoV-2 variants on stainless steel coupons up to 48 hours were similar (*P* > 0.05; Fig. 3A). However, a difference was observed at 24 hours at which WA-1 and Delta exhibited infectivity reductions of 0.8 and 1.0 log, respectively, while the infectivity of Omicron dropped more than 2.3 log to undetectable levels. At 48 hours PI, the infectivity of both WA-1 and Delta was undetectable as they dropped by more than 2.3 log. On glass coupons, however, WA-1 and Omicron lost viability through 48 hours, while Delta remained viable until 72 hours (Fig. 4A). Regardless of coupon type or virus variant, the difference in viral RNA titers was less than 3 Ct values (Fig. 3B and 4B). Comparing the persistence of the SARS-CoV-2 variants with OC43 and BoCoV, there was no significant difference in their persistence on stainless steel coupons (*P* > 0.05). On glass coupons, the persistence of OC43 and the SARS-CoV-2 variants did not significantly differ either (*P* = 0.180).

## DISCUSSION

We selected three RG2 human coronaviruses (OC43, 229E, and NL63) and one bovine coronavirus (BoCoV) as candidate surrogates to determine the environmental persistence of SARS-CoV-2. We evaluated and optimized the recommended cell culture assays for all four viruses, and for OC43, HRT-18G cells showed improved sensitivity and reduced the incubation time to reach complete CPE from 7 to 5 days. In contrast, at day 7 PI, the alpha coronaviruses (229E and NL63) manifested delayed CPE or no CPE at all, specifically with low-titer virus inoculum. This observation is well supported by previous reports of poor assay sensitivity using MRC5 and LLC-MK2 cell lines for 229E and NL63, respectively (23, 24). For 229E, MRC-5 cells presented cultivation challenges such as slow virus growth and high nutrient requirements, likely decreasing assay sensitivity, as has been reported previously (24). However, overall, the host cell lines selected on the basis of American Type Culture Collection (ATCC) guidelines and published research (1, 18, 23) for each of the four BSL-2 level coronaviruses are effective for virus propagation, but the host cell lines for 229E and NL63 are not sensitive enough to accurately measure infectious virus. Hence, we selected OC43 and BoCoV and compared the environmental persistence on different hard surfaces of these viruses with the three SARS-CoV-2 variants (WA-1, Delta, and Omicron).

Despite their high genetic similarity (95%) with SARS-CoV-2 and use of the same cell receptor (9-O-acetylated sialo glycans) (37, 38), the environmental stability of BoCoV and OC43 on the different hard surfaces was quite different. This discrepancy suggests that genetic factors alone may not fully predict surface stability, pointing to the complexity of viral environmental resilience. For the three SARS-CoV-2 variants, the duration of viability on coupons was similar, as was reported previously (39). However, we noted slight variations in the level of viability reduction during the first 48 hours. A possible explanation for these variations may be that, although all three variants were prepared and tested under similar conditions (e.g., inoculum titer), they may differ in the proportion of pseudoviruses or quasispecies within the virus stock (40), potentially explaining this variation in viability. Additionally, due to the limited number of test samples, the results may not be robust, and more data are required to better assess variability among viruses.

OC43 and BoCoV have been used by several research groups as surrogate models to study the persistence of SARS-CoV-2 (15–19, 21). Our data further support the utility of both RG-2 level viruses, highlighting the convenience and high sensitivity of cell culture assays for these viruses. Nevertheless, our study emphasizes the challenges of direct comparison as the persistence on stainless steel of neither OC43 nor BoCoV fully mirrors that of the SARS-CoV-2 variants, although OC43 showed the closest resemblance on glass coupons. Admittedly, different inoculum titers between surrogate and SARS-CoV-2 variants may influence the stability of these viruses on surfaces, affecting our interpretation. However, considering that larger quantities of virus are more likely to persist longer, the rapid decline in OC43 relative to SARS-CoV-2 variants also indicates that OC43 is less stable on surfaces. Overall, the observed distinct surface persistence characteristics between OC43 and BoCoV also warrant caution when extrapolating these findings to SARS-CoV-2, especially, in the absence of human exposure data. Additionally, our findings confirm that, while the reduction in viability varies by virus type and surface material, viral RNA of SARS-CoV-2 variants and surrogate viruses can still be detected on surfaces for extended periods of time (4, 5). This underscores the importance of using caution when interpreting the results of studies that rely on the detection of viral RNA as a measure of fomite transmission risk.

Our data are in agreement with previous studies indicating that surface composition significantly affects coronavirus inactivation (10, 16, 41–45). Specifically, while non-porous materials like plastic and stainless steel generally support longer virus viability than porous materials like cardboard (45), our findings of similar persistence between brushed stainless-steel surfaces and plastic may contradict expectations regarding the behavior of smooth surfaces. Notably, touchscreen glass surfaces facilitated more gradual virus inactivation than other non-porous materials such as stainless steel and vinyl, corroborating the observations on SARS-CoV-2 (41). This suggests that factors other than surface texture may be important. Specifically, on non-porous or hydrophobic surfaces such as touchscreen glass, droplets may dry in a more aggregated form, potentially impacting virus survivability. Conversely, on vinyl surfaces, we observed a more rapid inactivation of OC43 and BoCoV. Recent research by Watanabe et al. (16) highlighted the reduced stability of SARS-CoV-2 and BoCoV on materials such as nitrile rubber and soft polyvinyl chloride. Hence, potential interactions with various chemical additives in these materials may accelerate virus inactivation on such surfaces. Overall, our data underscore the complex interplay between surface composition and virus inactivation, highlighting the need to at least consider material-specific considerations in cleaning protocols and transmission risk assessments in diverse environments.

Our study demonstrated that OC43 and BoCoV are practical surrogates for SARS-CoV-2. However, these viruses exhibit distinct persistence characteristics that do not fully mimic SARS-CoV-2. Therefore, we suggest that OC43 and BoCoV may serve as RG-2 surrogates for SARS-CoV-2 under specific conditions, but caution should be applied when generalizing these results. Additional studies could be considered including evaluations of other candidate surrogate viruses such as porcine epidemic diarrhea virus, phi6 bacteriophage, and MHV (11–13). More importantly, numerous factors, including temperature, humidity, and virus titer, influence viral stability. To draw conclusive findings, additional variables affecting viral stability must be considered. Additionally, the link between environmental contamination by SARS-CoV-2 and the discharge of bodily fluids from infected individuals (46), coupled with the enhanced stability of the virus in these bodily fluids (47, 48) underscores the need for additional research.
